# Impact of examined lymph node number on lymph node status and prognosis in FIGO stage IB-IIA cervical squamous cell carcinoma: A population-based study

**DOI:** 10.3389/fonc.2022.994105

**Published:** 2022-09-20

**Authors:** Jiahui Yong, Baicheng Ding, Yaqin Dong, Mingwei Yang

**Affiliations:** ^1^ Department of Transfusion, The First Affiliated Hospital of University of Science and Technology of China, Division of Life Sciences and Medicine, University of Science and Technology of China, Hefei, China; ^2^ Department of Emergency Surgery, The First Affiliated Hospital of Anhui Medical University, Hefei, China; ^3^ Department of Radiation Oncology, The First Affiliated Hospital of Anhui Medical University, Hefei, China

**Keywords:** lymphadenectomy, hysterectomy, examined lymph node count, prognosis, SEER, cervical squamous cell carcinomas

## Abstract

**Objective:**

We aimed to investigate the association of examined lymph node (ELN) number with lymph node status and long-term survival in FIGO stage IB-IIA cervical squamous cell carcinoma(CSCC) and to determine the minimum number of ELN associated with survival improvement.

**Method:**

Data from the Surveillance, Epidemiology, and End Results Program (SEER) database of FIGO stage IB-IIA CSCC patients undergoing hysterectomy and pelvic lymphadenectomy in 2004-2016 were analyzed to explore the relationship between ELN number and lymph node status and overall survival (OS) by using the multivariable approach. The estimated probability of falsely identifying a patient as node-negative and the hazard ratios (HRs) for each ELN was fitted with a LOWESS smoother, and the structural breakpoints were determined. X-tile software was used to determine the optimal cutoff value for ELNs.

**Results:**

A total of 2627 patients were analyzed. The optimal cutoff value of the ELN number was identified as 7 based on the results of X-tile software. The structural breakpoints according to the associations between the number of ELNs and the estimated risk of false-negative lymph node dissection and HRs for overall survival were 9 and 8, respectively. The multivariate analysis indicated that ELN number was an independent prognostic factor for OS, both as a continuous or categorical variable. To further explore the effect of more ELNs on survival, another cutoff value of 17 was chosen to compare the survival curves of patients. The multivariate-adjusted COX model showed that patients with ELN<8 had a significantly higher risk of death than those with ELN8-17 (HR=1.447, 95% CI =1.075-1.947, *p*=0.015), but there was no significant difference in overall survival between patients with ELN>17 and patients with ELN8-17 (HR=0.822, 95%CI =0.665-1.016, *p*=0.070).

**Conclusion:**

A sufficient number of ELNs was associated with better long-term survival in FIGO stage IB-IIA CSCC. At least 8 ELNs need to be examined for prognostic stratification. Excessive lymph node dissection (ELN>17) may not confer additional survival benefits.

## Introduction

Cervical cancer is the fourth most frequently diagnosed cancer and the fourth leading cause of cancer death among women, posing a serious threat to women’s health (1). Different from previous staging systems, the 2018 International Federation of Gynecology and Obstetrics (FIGO) staging system incorporates lymph node status into staging, emphasizing the role of lymph node metastasis in tumor progression and prognosis (2, 3). Pelvic lymphadenectomy is an essential procedure for the surgical treatment of early-stage cervical cancer (4). Several retrospective reports have demonstrated improved survival mostly for patients after debulking of grossly involved nodes (5). However, considering the low rate of pelvic lymph node metastasis in early cervical cancer (6), unnecessary lymph node dissection may also lead to increased postoperative complications, such as pelvic lymphocyst and lower extremity lymphedema, which can seriously affect the quality of life of patients (7). Given the clinical potential of lymph nodes in anti-cancer immunity, negative lymph nodes should also be preserved (8).

Many large studies of colon, breast, ovarian, and endometrial cancers have shown a positive association between the number of removed lymph nodes and prognosis (9–12). However, it remains controversial whether detecting more lymph nodes provides a survival benefit in early-stage cervical cancer treated initially with surgery (13–15) and whether a minimum number of lymph nodes should be considered in lymph node dissection.

To address these outstanding questions, we used data from the Surveillance, Epidemiology and End Results (SEER), a large publicly available database, to analyze the number of lymph nodes examined in relation to lymph node status and overall survival, and to further determine the minimum number of lymph nodes to be examined associated with survival improvement. Given the different biological behavior underlying various tumor characteristics may affect the prognostic significance of examined lymph node (ELN), we limited the study to squamous cell carcinoma (SCC).

## Material and method

### Patients

The information of the patients was obtained from the SEER program which covers nearly 28% of the cancer patients in the United State and is publicly available and de-identified (16).

Patients diagnosed with FIGO stage IB–IIA (according to the 2009 FIGO staging system) cervical squamous cell carcinoma who underwent hysterectomy and lymphadenectomy with at least one examined LN between 2004 and 2016 were included in the study. We excluded patients diagnosed under 18 years old or over 80 years old, with the previous history of cancer, with unknown ELN, PLN, and clinical features, who had received neoadjuvant chemotherapy or preoperative radiotherapy or died within 1 month after surgery to reduce the impact of perioperative events on survival.

### Statistical analysis

The linear regression analysis was applied for investigating the relationship between the number of positive lymph nodes (PLNs) and ELNs. Stage migration was assessed by correlating ELN number and the proportion of positive versus negative nodal status using binomial logistic regression models, with adjustment for potential confounders associated with ELN or PLN number before or during surgery. The association of ELN number with overall survival was investigated and visualized using Cox proportional hazards regression models, with adjustment for other significant prognostic factors. Sensitivity analyses were performed by stratifying the models by demographic, clinical, and pathologic characteristics. Interaction tests were performed to evaluate associations between ELN numbers and the stratification factors.

To estimate the risk of false-negative LN dissection, the distribution of the percentages of positive metastatic LNs among all patients with at least 1 positive LN was fitted using a beta-binomial distribution according to the mathematical model proposed by Robinson et al. (17) and used the resulting model parameters to estimate the probability of having no positive LNs observed in truly node-positive disease. The estimated probability of falsely identifying a patient as node-negative and the hazard ratios (HRs) for the survival of each ELN number compared with the most frequent ELN number (as a reference) was visualized using the locally weighted scatterplot smoothing (LOWESS) smoother with a bandwidth of 2/3. Structural breakpoints were determined by ‘strucchange’ packages in R version 4.1.0 (The R Foundation for Statistical Computing, Vienna, Austria). The breakpoints were deemed as the threshold of clinical impact. X-tile software version 3.6.111 was applied to explore the ideal cut-off value of the ELN number for the largest OS difference. All statistical analyses were carried out using R software and X-tile software. *P* <.05 was considered statistically significant. Continuous variables are shown as median with interquartile range (IQR). Categorical variables are expressed as percentages.

## Result

### Patient characteristics and distribution of ELNs

**Figure 1:**
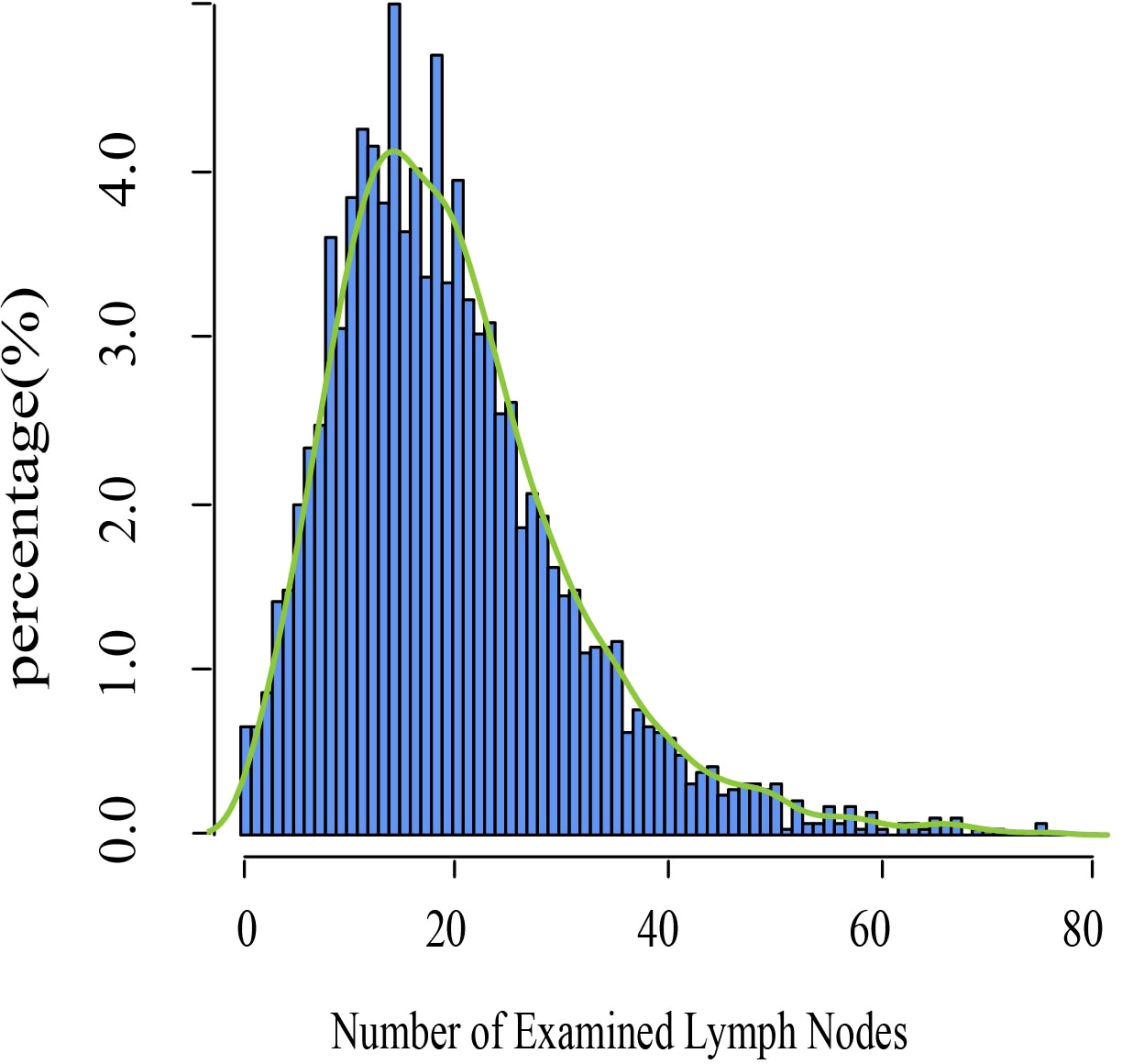
Distribution of the number of examined lymph nodes in the SEER database.

**Figure 2:**
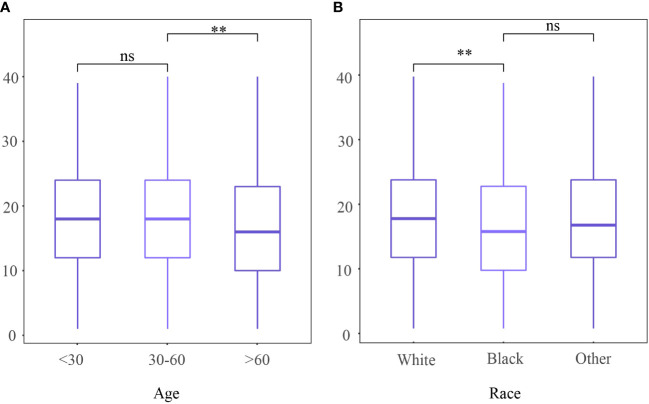
Number of examined lymph nodes among different **(A)** age and **(B)** race. ns,no significant difference; **P <0.01.

A total of 2627 patients were eligible for analysis. **Table 1** shows the baseline characteristics of the patients. The median age of the patients was 43 years (range,20-80 years), and about 76.5% of the participants were of white ethnicity. Most patients were classed as stage IB1- IB2 (76.7%). Radical hysterectomy was the most common surgical procedure (64.1%). Most cancers were moderately differentiated or poorly differentiated/undifferentiated (46.5%,49.8%, respectively). The incidence of lymph node metastases was 20.7%. During a median follow-up of 99 months, 412 deaths were recorded (censored, 84.3%). The distribution of ELN numbers is shown in **Figure 1**. we restricted the range of the studied ELNs to 1-40 as it is closer to the normal distribution and clinical practice. The most frequent number of ELNs in the studied cohort was 15, with a median of 17 (IQR 12–24). Notably, the mean number of ELNs differed significantly within subgroups of age and race in the entire cohort (**Figure 2**). Considering OS as the endpoint, the optimal cut-off value of ELNs was determined using X-tile software as 7, and patients were then divided into two groups with an ELN <8 or ≥ 8 for subsequent analysis.

**Table 1:** Baseline clinicopathological characteristics.

Characteristic		N (percentage)Or Median (25%-75%)
**Age (years)**	As continuous	43 (36-53)
	<30	194 (7.4)
	30-60	2068 (78.7)
	>60	365 (13.9)
**Year of diagnosis**	2004-2009	1310 (49.9)
	2010-2016	1317 (50.1)
**Race**	White	2010 (76.5)
	Black	300 (11.4)
	Other	317 (12.1)
**Marital status**	Married	1294 (49.3)
	Single	790 (30.1)
	Other	543 (20.7)
**Tumor location**	Cervix uteri	2258 (86.0)
	Endocervix	212 (8.1)
	Exocervix	64 (2.4)
	Overlapping lesion of cervix uteri	93 (3.5)
**Differentiation**	Well	96 (3.7)
	Moderately	1222 (46.5)
	Poor/undifferentiated	1309 (49.8)
**FIGO stage**		
	IB1	2014 (76.7)
	IB2	415 (15.8)
	IIA1	123 (4.7)
	IIA2	75 (2.9)
**Examined lymph node count**	As continuous	17.0 (12.0-24.0)
**Positive lymph node count**	As continuous	0 (0-0)
**Pathologic N stage**	N0	2084 (79.3)
	N1	543 (20.7)
**Tumor size, mm**	As continuous	25.0 (15.0-38.0)
**Resection type**	Radical	1684 (64.1)
	Simple/pan	737 (28.1)
	Not otherwise specified	206 (7.8)
**chemotherapy**	Yes	750 (28.5)
	No/unknown	1877 (71.5)
**radiotherapy**	Yes	1133 (43.1)
	No/unknown	1494 (56.9)

### Number of examined lymph nodes and lymph node status

Linear regression analysis showed a weak positive correlation between ELN number and PLN number (R^2^ = 0.002, *P* = 0.043), and the relationship was not significantly improved when restricted to patients with node-positive disease (R^2^ = 0.011, *P*=0.016). After adjustment for potential confounders, including age, year of diagnosis, race, tumor location, differentiation, tumor size, resection type, and FIGO stage, there was no correlation between the number of ELNs and the odds for negative-to-positive node stage migration in both overall (OR=1.000, 95% CI =0.989–1.011, *P*=0.997) and in subgroups of age group, race, differentiation, FIGO stage, and resection type (**Table 2**).

**Table 2:** Association of examined lymph node number (as a continuous variable) with negative-to-positive node stage migration.

Stratification	Adj. OR∗	95% CI	*P*OR	*P* interaction
**Overall**	1.000	0.989-1.011	0.997	
**Age group**				0.400
<40	0.998	0.980-1.016	0.815	
40-60	0.998	0.982-1.014	0.795	
>60	1.018	0.988-1.050	0.245	
**Race**				0.683
White	0.998	0.985-1.010	0.707	
Black	1.007	0.971-1.045	0.693	
Other	1.008	0.977-1.040	0.613	
**Differentiation**				0.988
Well	1.014	0.917-1.121	0.782	
Moderately	1.001	0.985-1.018	0.881	
Poor/undifferentiated	1.000	0.985-1.015	0.991	
**FIGO stage**				0.967
IB1	1.001	0.988-1.015	0.841	
IB2	0.996	0.974-1.019	0.762	
IIA1	0.983	0.935-1.034	0.508	
IIA2	1.008	0.945-1.074	0.814	
**Resection type**				0.891
Simple/pan	1.005	0.983-1.027	0.670	
Radical	1.001	0.986-1.014	0.967	
Not otherwise specified	0.989	0.950-1.029	0.580	

Adj., adjusted; OR, odds ratio.

**Figure 3:**
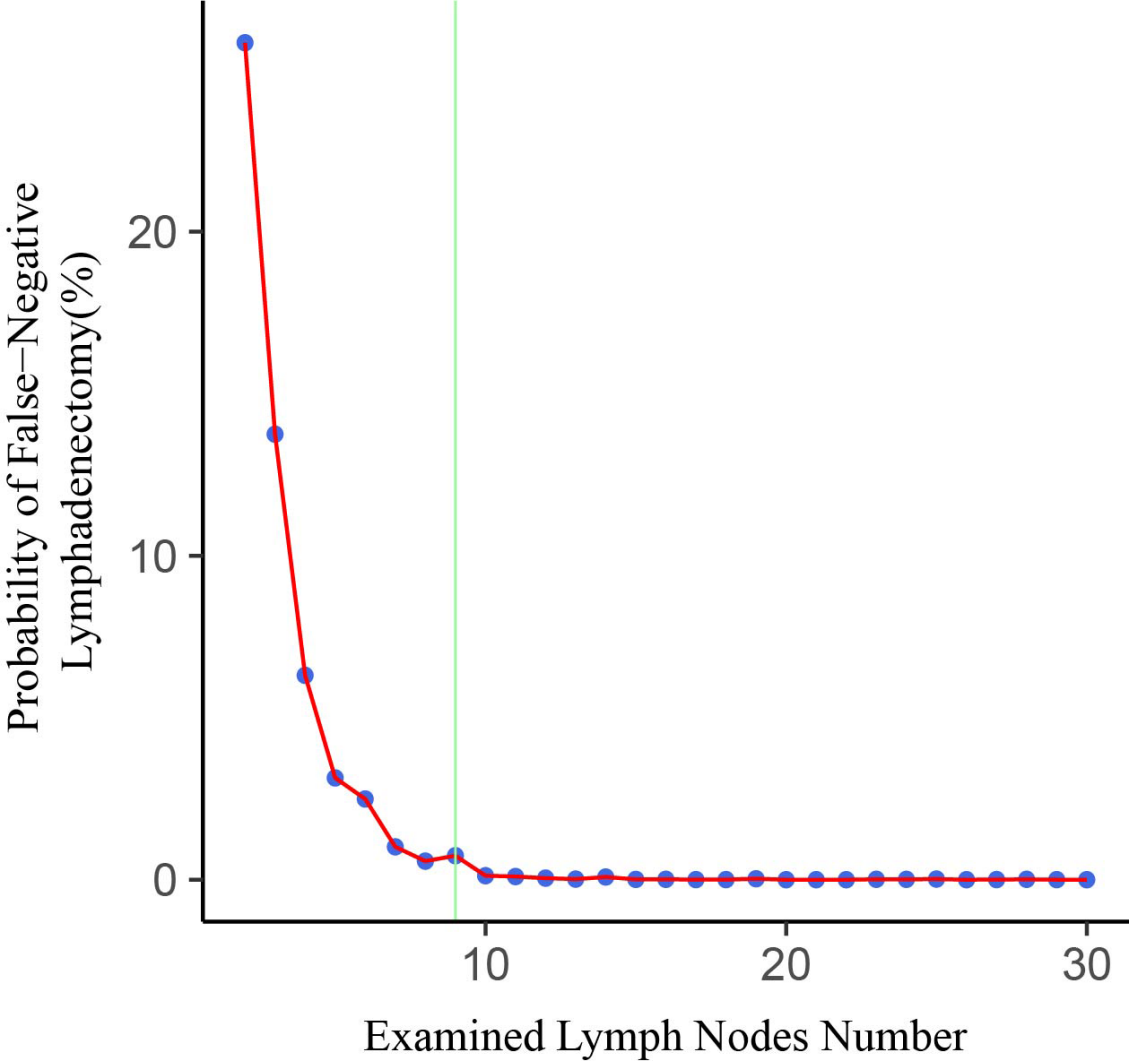
Association of examined lymph node (ELN) number with probability of undetected positive lymph nodes in a patient with truly LN-positive disease. The structural breakpoint determined by using ‘strucchange’ packages in R is shown in green.

The fitting curve and corresponding structural break point of ELN number with the estimated probability of false-negative disease are shown in **Figure 3**. It is observed that the estimated false negative rate drops rapidly before the ELN count is 9, after which the probability approaches 0.

### Number of examined lymph nodes and overall survival

The prognostic impact of ELN number was assessed using multivariable Cox proportional hazards regression models. After multivariable adjustment for the other prognostic factors, including age, year of diagnosis, race, tumor location, tumor size, differentiation, FIGO stage, PLN number, resection type, administration of radiotherapy and chemotherapy, and marital status, the results showed that ELN number was an independent prognostic factor for OS as a continuous or a categorical variable (**Table 3**).

**Table 3:** Multivariable Cox proportional hazard regression analyses of factors associated with overall survival of all patients.

Characteristic	HR	95%CI	*P*
**Age (continuous variable)**	1.019	1.010-1.027	<0.001
**year of diagnosis**			
2004-2009	1		
2010-2016	0.970	0.786-1.198	0.970
**Race**			
White	1		
Black	1.377	1.043-1.819	0.024
Other	0.933	0.683-1.275	0.665
**Marital status**			
Married	1		
Single	1.010	0.796-1.282	0.935
Other	1.164	0.907-1.494	0.234
**Tumor location**			
Cervix uteri	1		
Endocervix	0.616	0.406-0.934	0.023
Exocervix	0.562	0.250-1.263	0.562
Overlapping lesion of cervix uteri	0.869	0.508-1.489	0.610
**Differentiation**			
Poor/undifferentiated	1		
Moderately	0.951	0.501-1.808	0.879
Well	0.906	0.743-1.106	0.333
**FIGO stage**			
IB1	1		
IB2	0.819	0.576-1.163	0.264
IIA1	1.698	1.175-2.455	0.005
IIA2	0.896	0.519-1.549	0.695
**Resection type**			
Radical	1		
Simple/pan	0.857	0.681-1.079	0.857
Not otherwise specified	1.564	1.151-2.125	0.004
**Radiotherapy**			
yes	1		
no	0.843	0.651-1.091	0.194
**Chemotherapy**			
yes	1		
no	0.971	0.747-1.263	0.971
**Positive lymph node count**	1.199	1.126-1.278	<0.001
**Tumor size**	1.030	1.021-1.038	<0.001
**Number of ELNs (n)**			
<8	1		
≥8	0.622	0.472-0.820	0.001
Number of ELNs (continuous variable)	0.988	0.977-0.999	0.037

We further tested the subgroup differences among various clinicopathological types and treatment factors by using interaction tests (**Table 4**). No significant subgroup differences were detected in the regression models, which means that a separate study of the association between ELN number and OS is not supported in these subgroups.

**Table 4:** Association of examined lymph node number (as a continuous variable) with overall survival in subgroups by clinicopathological types and treatment factors.

Stratification	Adj. HR∗	95% CI	*P*HR	*P* interaction
**Overall**	0.988	0.977-0.999	0.037	
**Age group**				0.329
<40	0.992	0.972-1.013	0.449	
40-60	0.993	0.977-1.010	0.418	
>60	0.968	0.943-0.993	0.013	
**Race**				0.736
White	0.991	0.978-1.004	0.190	
Black	0.979	0.951-1.008	0.158	
Other	0.986	0.953-1.020	0.404	
**Tumor location**				0.078
Cervix uteri	0.988	0.977-1.000	0.052	
Endocervix	1.043	0.991-1.098	0.108	
Exocervix	0.975	0.602-1.578	0.917	
Overlapping lesion of cervix uteri	0.869	0.772-0.978	0.019	
**Differentiation**				0.356
Well	0.920	0.811-1.045	0.200	
Moderately	0.987	0.971-1.004	0.142	
Poor/undifferentiated	0.991	0.976-1.006	0.260	
FIGO stage				0.752
IB1	0.984	0.969-0.999	0.035	
IB2	0.990	0.970-1.011	0.365	
IIA1	0.974	0.929-1.021	0.276	
IIA2	1.003	0.936-1.074	0.933	
**Resection type**				0.950
Simple/pan	0.988	0.965-1.011	0.301	
Radical	0.987	0.974-1.001	0.073	
Not otherwise specified	0.993	0.958-1.029	0.699	
**Radiotherapy**				0.268
yes	0.995	0.981-1.010	0.532	
no	0.980	0.962-0.997	0.023	
**Chemotherapy**				0.242
yes	0.997	0.979-1.014	0.702	
no	0.982	0.968-0.997	0.017	
**N stage**				0.396
node-negative	0.993	0.979-1.006	0.275	
node-positive	0.977	0.957-0.998	0.035	

Adj., adjusted; HR, hazard ratio.

**Figure 4:**
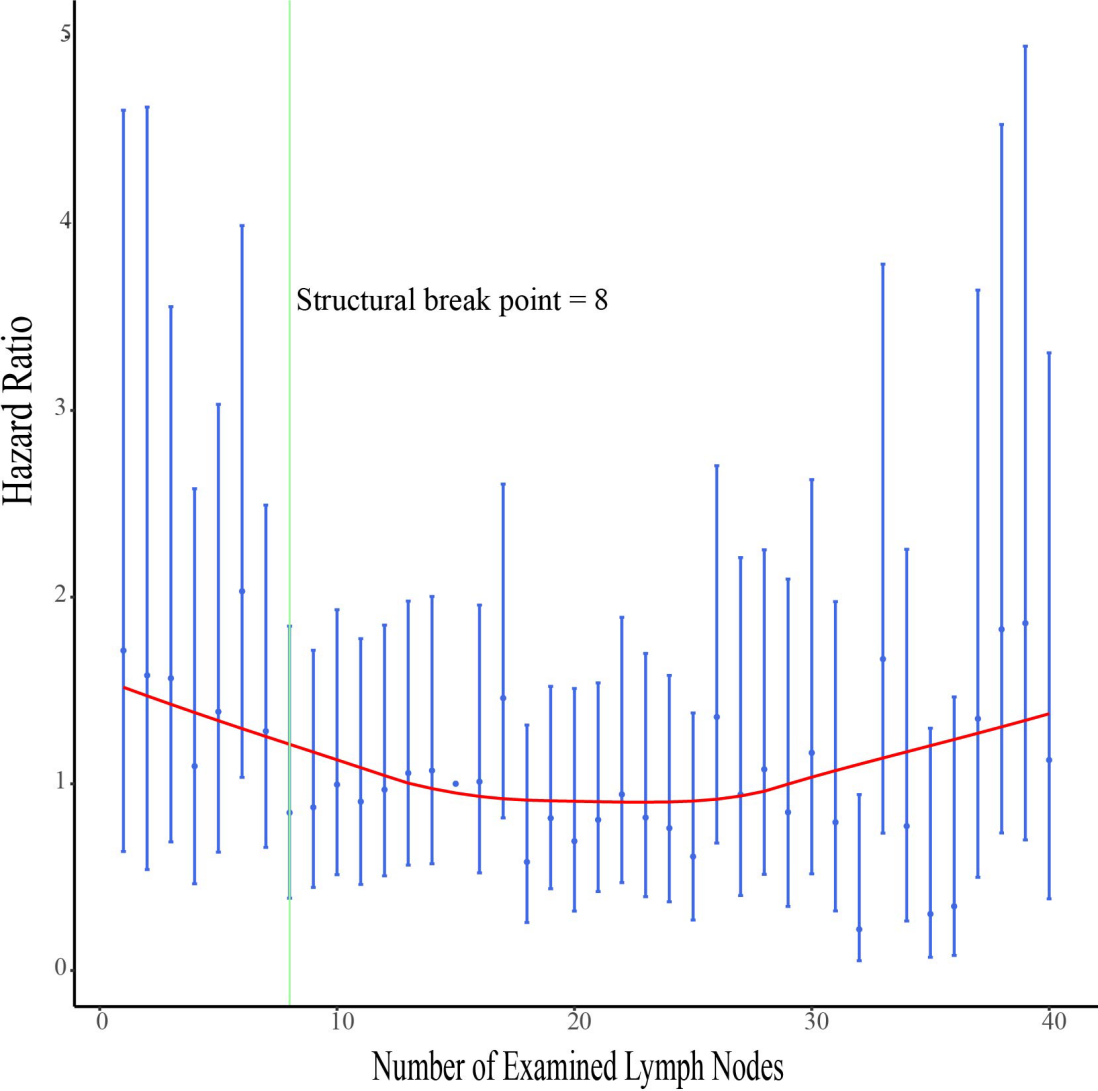
Association of examined lymph node (ELN) numbers with multivariable-adjusted hazard ratios (HRs) for overall survival. The adjusted HRs and the corresponding 95% confidence intervals are shown in blue, and the smoothed curves fitted using the LOcally WEighted Scatterplot Smoothing (LOWESS) method with a default bandwidth of 2/3 are shown in red. The structural breakpoint determined by using ‘strucchange’ packages in R is shown in green.

**Figure 5:**
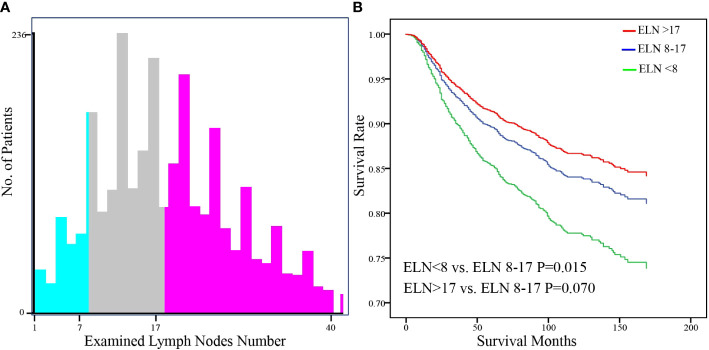
Identification of the optimal cut-off value of ELN for FIGO Stage IB-IIA Cervical Squamous Cell Carcinoma patients based on the x-tile software **(A)**. Stratification of adjusted overall survival according to cutoff values **(B)**. Multivariable Cox regression was used adjusting for age, year of diagnosis, race, tumor location, tumor size, differentiation, FIGO stage, PLN number, resection type, administration of radiotherapy and chemotherapy, and marital status.

The hazard ratios (HRs) for survival with each ELN are shown in **Figure 4**. The HR ratio markedly and rapidly decreased when the number of ELNs < 8, after which the curve flattened out and then gradually raised when the ELN number > 25. To further explore the effect of more ELNs on survival, another cutoff value of 17 was selected (**Figure 5A**). The multivariate-adjusted COX model showed that patients with ELN<8 had a significantly higher risk of death than those with ELN8-17 (HR=1.447, 95% CI =1.075-1.947, p=0.015; **Figure 5B**), but there was no significant difference in overall survival between patients with ELN>17 and patients with ELN8-17 (HR=0.822, 95%CI =0.665-1.016, p=0.070; **Figure 5B**).

## Discussion

Pelvic lymphadenectomy remains level I evidence for early-stage cervical carcinoma because lymph node status is difficult to assess with high accuracy on preoperative imaging or intraoperative sentinel lymph nodes (18). However, due to the low rate of lymph node metastasis in cervical cancer, the survival benefit of lymphadenectomy remains controversial. The survival benefit of lymph node dissection can be analyzed by two ways, direct survival benefit following resection of bulky positive metastatic lymph and indirect survival benefit due to proper disease staging and stage migration and tailored adjuvant therapy (19). A previous SEER study showed that more extensive lymphadenectomy (>30 nodes removed) improved survival in node-negative patients but had no effect on survival in node-positive patients (15). Peters et al. and Kim et al. found that the greater number of removed lymph nodes improved progression-free survival (PFS), especially in node-positive patients (13, 20). However, Guo et al. and ditto et al. did not observe any relationship between the number of removed lymph nodes and survival (21, 22). Zhou J et al. analyzed different histological subtypes of cervical cancer and showed that the number of ELNs could affect CSS and OS in patients with node-positive SCC patients but had no prognostic value in AC patients (23). This suggests that different biological behaviors behind different types of tumors may affect the prognostic significance of ELNs. In addition, the distribution of lymph nodes may also have an impact on the results. To homogenize the study, we included only patients with stage IB-IIA squamous cell carcinoma and excluded patients with stage IA2 because of their low rate of lymph node metastasis and unreliable tumor size (24). Considering actual clinical practice and normal distribution, ELN counts range from 1-40.

In this large retrospective study, we analyzed the association of ELN number with lymph node status and long-term survival in FIGO stage IB-IIA CSCC. Our results revealed that the number of ELNs was not associated with accurate staging after multivariate adjustment. However, examining a higher number of lymph nodes would reduce the estimated probability of undetected positive lymph nodes. The estimated false-negative rate approached 0 when the number of ELNs was greater than 9. Examining more than 9 lymph nodes may help to ensure the quality of the procedure. After multivariate adjustment, we observed that higher ELN counts were associated with better survival. A plausible hypothesis is that collecting more lymph nodes may facilitate a more complete resection of possible malignant remnants. Thus, patients with positive lymph node status are less likely to be tested negative, making these patients more likely to receive postoperative adjuvant therapy, which would improve long-term survival (25). In previous studies, many investigators have found that the number of ELNs has different effects on the prognosis of patients with different lymph node status. We discovered that the ELN number affected the results when used as a continuous or categorical variable. When ELN count was considered as a continuous variable, more extensive lymph node resection significantly improved the prognosis of patients with positive lymph nodes (HR=0.977, 95%CI= 0.9577-0.998, *P* =0.035), but had no effect on patients with negative lymph nodes (HR=0.993, 95%CI= 0.9799-1.006, *P*=0.275). However, when ELN count was used as a categorical variable (ELN <8, ELN≥8), the results were just the opposite. Removing more lymph nodes could significantly improve the prognosis of patients with negative lymph nodes (HR=0.629, 95%CI=0.452-0.877, *P*=0.006), but did not affect on lymph node-positive patients (HR=0.606, 95%CI=0.362-1.014, *P*=0.057). Therefore, we conducted interactive tests to compare the differences between groups, and the results showed that the ability to conduct separate studies based on lymph node status was insufficient. After further stratifying the population, we observed a significant positive correlation between the number of ELNs and OS in patients >60 years of age, with the overlapping lesion of cervix uteri, stage IB1, and not receiving radiation or chemotherapy. But Interaction tests also do not support the ability to separate these groups into separate studies.

The number of lymph nodes required to be examined in a lymphadenectomy for cervical cancer is not standardized. The range of the minimum number of lymph nodes for an adequate lymphadenectomy has been previously reported to be 10 to 25 (14, 26). The opinion of the European Organization for Research and Treatment of Cancer-Gynaecological Cancer Group (EORTC-GCG) is to remove at least 11 pelvic lymph nodes as surgical quality assurance (27). The International Union against Cancer proposed that to determine the status of lymph nodes, the minimum number of lymph nodes to be detected is 10 (28). By analyzing the correlation between ELN count and overall survival, we found that the minimum number of examined lymph nodes associated with survival improvement was 8. Based on the optimal cut-off value provided by x.tile software we divided the population into two groups for analysis, and multivariate analysis showed that patients with ELNs ≥8 had a better overall survival rate. To further explore the effect of a greater number of ELNs on survival, another cutoff value of 17 was chosen to compare the survival curves of patients with ELN>17 and patients with ELN8-17. The multivariate-adjusted COX model showed no significant difference between them. By visualizing the correlation analysis between the HR for survival and the number of ELNs, we also found a similar trend, the HR ratio markedly and rapidly decreased when the number of ELNs < 8, after which the curve flattened out, and gradually increased when the number of ELNs >25. A possible explanation is that more aggressive tumors are more likely to have more lymph nodes removed during surgery due to their increased lymph node size and number (29, 30).In addition, removing too many lymph nodes may lead to increased postoperative complications (5). Due to the limitations of the SEER database, we were unable to compare the biological differences behind the >17 ELNs group with that in 8-17 ELNs groups and the difference in the incidence of postoperative complications.

The current findings suggest an association between the number of examined lymph nodes and OS, but cannot infer a causal relationship. We need to emphasize that the ELN count is the total number of regional LNs removed by the surgeon intraoperatively and examined by the pathologist postoperatively. Therefore, the number of ELNs is influenced by other confounding factors, including the patient’s status, the skill of the operator, and the practice of the pathologist. Furthermore, there are limitations in the SEER database, such as the lack of information on LN sites and detailed clinicopathological characteristics. The European Organization for Cancer Research and Treatment-Gynecological Cancer Group agreed that each common iliac, external iliac, internal iliac, and obturator region must contain at least 1 lymph node examined (31). Therefore, more studies including information on the number of lymph nodes at each site, detailed tumor pathological characteristics, and patient recurrence information are needed to further validate our conclusion. In addition, considering that limited LN dissection may improve the quality of life, related research is also required.

## Conclusion

In summary, the number of examined lymph nodes is a significant prognostic factor in patients with FIGO stage IB-IIA CSCC. We believe that a minimum of 8 numbers of lymph nodes need to be examined to ensure better long-term survival for patients. Given that the number of lymph nodes examined >17 does not further improve survival, expanded lymph node dissection may not be necessary.

## Data availability statement

Publicly available datasets were analyzed in this study. The data can be accessed on SEER database (https://seer.cancer.gov/).

## Ethics statement

The SEER database is an open database available to all clinical researchers, and all data obtained are de-identified. In addition, all authors have signed the data-use agreement prior to accessing the raw data from the SEER database. And we followed the protocol throughout the study to protect the privacy of patients. Therefore no ethical review was required for this study.

## Author contributions

JY conceived the idea, designed the work, analyzed the data, and drafted the work. BD analyzed the data and performed the visualization. YD collected the data and participated in the revision. MY interpreted the data and supervised the study. All authors contributed to the article and approved the submitted version.

## Funding

This work was supported by Anhui Medical University Basic Medicine and Clinical Medicine Cooperation Research Promotion Program, Hefei, China [grant numbers: 2019xkjT025].

## Acknowledgments

We are very grateful to the staff of the Surveillance, Epidemiology, and End Results Program (SEER) for their excellent work in data collection and delivery.

## Conflict of interest

The authors declare that the research was conducted in the absence of any commercial or financial relationships that could be construed as a potential conflict of interest.

## Publisher’s note

All claims expressed in this article are solely those of the authors and do not necessarily represent those of their affiliated organizations, or those of the publisher, the editors and the reviewers. Any product that may be evaluated in this article, or claim that may be made by its manufacturer, is not guaranteed or endorsed by the publisher.
